# Low back pain patients' experiences of work modifications; a qualitative study

**DOI:** 10.1186/1471-2474-11-277

**Published:** 2010-12-06

**Authors:** Carol Coole, Paul J Watson, Avril Drummond

**Affiliations:** 1 Division of Rehabilitation and Ageing, School of Community Health Sciences, University of Nottingham, Queen's Medical Centre, Nottingham, UK; 2 Department of Health Sciences, Academic Unit, University of Leicester, Leicester, UK

## Abstract

**Background:**

Research indicates that work modifications can reduce sickness absence and work disability due to low back pain. However, there are few studies that have described modified work from the perspective of patients. A greater understanding of their experiences may inform future workplace management of employees with this condition.

**Methods:**

Individual semi-structured interviews were conducted with twenty-five employed patients who had been referred for back pain rehabilitation. All had expressed concern about their ability to work due to low back pain. Data was analysed thematically.

**Results:**

Many participants had made their own work modifications, which were guided by the extent of control they had over their hours and duties, colleague support, and their own beliefs and attitudes about working with back pain. A minority of the participants had received advice or support with work modifications through occupational health. Access to these services was limited and usually followed lengthy sickness absence. Implementation largely rested with the manager and over-cautious approaches were common.

**Conclusions:**

There was little evidence of compliance with occupational health guidance on modified work. There appears to be insufficient expertise among managers and occupational health in modifying work for employees with low back pain and little indication of joint planning. On the whole, workers make their own modifications, or arrange them informally with their manager and colleagues, but remain concerned about working with back pain. More effective and appropriate application of modifications may increase employees' confidence in their ability to work.

## Background

Low back pain is a major cause of sickness absence and work disability. The majority of workers who take sick leave due to back pain return to work quickly, but many will continue to experience pain [1] and between 18% and 44% will have a further episode of absence within the year [2]. Previous studies have described the occupational factors that contribute to work disability as 'blue' and 'black' flags [3]. Blue flags refer to workers' perceptions of their work and workplace that can impede recovery, for example a belief that work is harmful. Black flags are actual workplace or work-related influences, for example managers' negative attitudes. Shaw and colleagues [3] have recently identified seven of these flags as 'core' workplace variables to be used in the assessment of occupational obstacles, one being the ability to modify or adjust work. In the UK, a recent review [4] urged that workplaces 'consider potential adjustments which could enable employees to remain in or return to work while recovering from ill-health'.

Work modifications may include changes to the workplace, equipment, work design and organisation, working conditions and/or work environment [5]. There is evidence that modified work can reduce or avoid sickness absence, increase return to work rates and job retention and decrease the recurrence of symptoms [6-9]. Modifications should be temporary; the aim should be for a gradual return to normal duties.

Advice on the management of workers with musculoskeletal disorders is available to employers in the UK through the Health and Safety Executive [10] and the Faculty of Occupational Medicine [11]. In addition to the availability of modified work, these also support early reporting of symptoms, prompt assessment and management of obstacles to recovery, and good communication between the 'key players'. More recently, combined clinical and occupational guidance has been published [12]. Previous research has identified that the involvement of clinicians and therapists in advising on work modifications can be beneficial [13]. However, it is not known if such approaches are common in the UK nor which employers implement them, and there is little evidence that UK General Practitioners (GPs) and clinicians are involved. Current research suggests the structures to support joint working do not exist [4,14,15]. Responsibility therefore lies with the employer, employee, and any occupational health service provided by the employer (the latter are not routinely available in the UK).

Although there has been some quantitative research conducted into workplace interventions for low back pain, there is little from the patient perspective. A greater understanding of experiences in attempting to remain at work with back pain may inform future workplace management of this condition. The aim of this study was therefore to explore employed patients' experiences and perceptions of work, prior to attending a rehabilitation programme.

## Methods

### Research design, sample and recruitment

Data were collected through individual semi-structured interviews with a convenience sample of low back pain patients who had been referred to multidisciplinary rehabilitation. The purpose of the interview was to enable each participant to report their individual experience of working with back pain.

Participants were recruited to the study by clinicians from a multidisciplinary back pain rehabilitation team during routine initial assessment, following referral by the patient's GP or other healthcare professional. The eligibility criteria were the participant was 1) fluent in English 2) had been offered a programme of rehabilitation and 3) had responded positively to a written statement 'I am concerned about my ability to work due to low back pain' completed at the start of their initial assessment.

A total of 25 patients participated in the study. Thirteen were female, twelve male. They represented both private and public sector; professional, skilled, semi-skilled and unskilled workers, manual and non-manual occupations. Twenty worked for large enterprises (>250 employees), three worked for small enterprises (<50 employees) and two were self-employed. Of the twenty who were employed by large enterprises, ten worked in the private sector, ten in the public sector. Six were off sick at the time of the interview. Six had never taken sick leave for back pain and eleven had taken no sick leave for back pain in the previous six months. The mean age was 44.7 yrs (range 22-58 yrs), and mean history of back pain 6.8 yrs (range 3 mths-35 yrs). Further detail of the research design has been published elsewhere [16].

Ethical approval was granted by the Nottingham 1 Research Ethics Committee.

### Data collection

Interviews took place during July and August 2008, either at the participant's home, workplace, or at the office base of the rehabilitation team. Interviews lasted approximately 45 minutes and were digitally recorded. Written consent was obtained at the interview.

A list of topic areas was developed through a review of the literature, by discussion with two user representatives, and between the authors. Topics included the experience of working with back pain and the help received in managing their symptoms at work and in remaining at work. The list was prepared as a guide for the interviews to ensure that the same topics were covered in each, but open questions allowed participants to add further individual experiences and observations; amendments and additions to the guide were made in response to new topics arising as the interviews progressed.

### Data analysis

All of the interviews were conducted and recorded by the interviewer and transcribed verbatim. A qualitative software package NVivo8, (QSR International Pty Ltd) was used to manage the data which were analysed thematically [17]. Initial codes were refined following constant comparison of the transcripts. Themes were identified and analysed by repeated study of the scripts and discussion with the research team as the data collection proceeded. Two of the researchers (CC and PJW) then reviewed and agreed the themes and findings.

## Results

A number of themes and sub-themes were identified, as shown in Table 1. These themes are illustrated in the text with quotations from the participants.

**Table 1:** Main themes and subthemes identified through analysis of the data.

Work modifications: Occupational Health assistance

A service for employers rather than employees

Advice may be over-cautious

Influence may vary and may be dependent on perceived causation

Modifications left to manager to implement

Work modifications: assistance from employers/managers

Help depends on the individual manager

May be over-cautious in their support

Managers with experience of back pain perceived to be more sympathetic

Work modifications: patient control

Easier to modify workload if in control

The pros and cons of working for oneself

Fewer options if working alone

Colleague support

### Work modifications: assistance from Occupational Health

#### A service for employers rather than employees

The majority of the participants in this study worked for large employers (>250 employees) who are more likely to provide an occupational health service. Some of those who had accessed occupational health reported positive experiences and examples of practice which reflected current occupational guidelines [10-12]. However, several participants were unsure whether there was such a service, or what it might offer them. It was usually accessed through referral by (and at the discretion of) the line manager; a service that the employee might be 'sent to' or that the employer was 'willing' for the employee to access. Agreeing to attend was seen as a necessary procedure to be followed, for example:

'I've been to occupational health at work, and basically been compliant throughout the whole thing.' (10 Male)

The view that occupational health was employer-orientated could result in a lack of trust. Employees might have doubts over confidentiality, or whether it might affect their job security if a judgement was made that they were not fit to work. This participant describes why she had chosen not to use a telephone help-line:

'It says that it's private and confidential, but I do know for a fact that it goes back to your managers. Which to me is wrong.' (25 Female)

Occupational health was generally perceived as an absence management procedure, associated with 'return to work' interviews following a period of sick leave, rather than a service for supporting people to remain at work. Two participants on sick leave for six weeks at the time of the interview expected to be referred for their first consultation on their return, not before.

In the time between her clinical assessment and the study interview the following participant had been retired on ill health by her employers, having been off sick for a year. Modified work did not appear to have been considered by occupational health, despite her motivation to return to work:

'Well, I had to go and see a private doctor from XXXX and then another one. I had to see two separate ones. And they did all the same that everyone else has done. They all say that it wouldn't be advisable for me to do the job... probably they are right because I'm still a little bit nervous in case that goes again. Nobody's ever told me it won't, you know, so I suppose.. if it did happen, and they'd let me go back to work - everybody's frightened of suing. I think I could have probably gone back to it myself...' (18 Female)

Of those who had received consultations, these were generally conducted away from the work-site. Only one participant described a visit by an occupational physician to look at his work environment; another participant, a staff nurse, had been promised a visit but it had not taken place. Assessment of participants' ability to do their job was generally through discussion rather than observation; some also reported a limited test of physical function such as bending. One participant questioned the validity of an assessment which had been conducted by telephone.

#### Advice may be over-cautious

From the descriptions that participants gave, the advice received as a result of the consultations varied in its adherence to occupational guidelines [10-12]. Guidelines recommend temporary modifications to enable a graded return to normal duties, without raising fears about further pain, or causing 'damage' to the back. However it seemed that some participants had been advised to avoid certain duties rather than being gradually exposed to normal activities. This was particularly the case in tasks thought to be more closely associated with back pain, and where back pain was perceived to have started at work.

Two participants had become involved with Occupational Health following accidents at work. One had had to contact them himself, but appreciated their support and found them effective in advising him on a phased return to full duties, although the underlying message seemed to be that he should be careful about lifting and six months later he was still on 'light' duties:

'Occupational Health came in at work, and they said "no, don't do anything, just do 'light' duties" - you know - computer stuff - recommended to HR what I should do and things - and said keep on light duties for another - month I think he said and then he's going to come in and assess the jobs and things and say whether or not he thinks that they're suitable.' (6 Male)

The other participant was still on 'light' duties over a year later and was in the process of applying for disability benefits. In her case their advice on modifications had also helped her remain in employment, but not return to full duties, and implementation seemed to largely rest with her manager.

Another participant had referred herself to Occupational Health as she was keen to return to work after four months sick leave. Their advice on modifications had helped her to return to work, but again, implementation of their advice largely rested with the manager, and advised restricted lifting with no apparent indication of when this arrangement should change:

'It's like I had to refer myself to OH - and that's the only time I've got anywhere. They said I am better off going back to work if at all possible. They actually wrote and said that I should be fine going back to work, part-time........I'm not allowed to pick - 10 kg or something - the (occupational health) doctor put it on the letter.' (26 Female)

Less common was the experience this participant reported by a care worker who had felt reassured by her consultation that modifications were not required:

*'I saw (occupational health physician) and you know he talked through everything with me, examined me, and he wrote a letter to my manager and sent me a copy, and said that I could carry on with normal work activities. He felt that my back wouldn't stop me doing anything, but if I did something to aggravate it, it wouldn't make it worse - I'd just be in extra pain for a few days*.'*(5 Female)*

However, this participant had taken minimal sick leave for her back pain. She had been given this advice through a consultation triggered by a period of 8 weeks sick leave due to depression, not back pain.

### Influence may vary and may be dependent on perceived causation

There were different experiences in the extent to which the advice of occupational health would be taken up by employers. For example, this participant had been on sick leave for over six months as she was struggling to drive to work because of her back pain. She had attended more than one occupational health consultation. Her public sector employers were either unable or unwilling to act on the advice they had been given. At the time of the interview she was involved in legal proceedings against her employers over the application of reasonable adjustments as defined by the Disability Discrimination Act [18] and had been off sick for seven months. She describes one of the consultations:

'He said "the sensible solution would be to relocate her to an office closer to her home, otherwise the problem will not go away". That was the opinion. I had a meeting briefly after the report and she (her manager) said "well, there's nothing I can do, there's no jobs there". Problem is, employers can just ignore what they say - and they have done, throughout the whole of this.' (12 Female)

The two participants whose back pain had followed workplace accidents indicated that the response of their employers was associated with the perceived cause of their pain:

*'But he's been actually pretty good (Occupational Health Physician) - he's given quite good advice I think and given them a bit of a kick as well actually - when he came in he just looked around and said "Oh God that's awful - you shouldn't be doing that" - and took the boss into the office - and it was quite nice really that there was.. '*There was someone looking out for you?' *Yes.' (6 Male)*

And

'I think they're more understandable (understanding) because it's been done at work. They're more lenient. I think if I'd have done this - say I was gardening at home and I'd done it, then I think they'd have been more inclined to have let you go, more so than try and help you to work through it.' (24 Female)

### Modifications left to manager to implement

It appeared rare for occupational health personnel to meet with anyone other than the patient. Usually the patient was left to act as a conduit between occupational health, their employer and their GP. In the two instances where workplace injuries were perceived to have taken place, occupational health did meet with the manager/employer; otherwise communication between the parties was by written report or letter. However written communication does not provide an opportunity for all those involved to clarify or discuss any advice or recommendations given, and how it might be implemented or evaluated. This participant described how there seemed to be an expectation from both the physician and the manager that the participant was responsible for the transmission of information:

*'He said "email me your latest thing from the last meeting you had, I'll look at it, review it and then forward it to her (the manager*) *and then she can read that then she's got everything there". So he was - I think he's of the opinion- that he wants to check what I'm saying and make sure that things are recommended correctly, rather than them asking me, I say something and it goes wrong. So I said "the best answer is to go through the right channel".' (6 Male)*

This participant highlights a similar lack of clear, effective communication:

'I haven't been back to see her since that initial consultation. It was a series of consultations and on one of them the boss wanted to sit in, I had no objection, but the Occupational Health officer did, so - it didn't happen and the boss wasn't pleased about that and she basically gave me a good grilling - "well, what did she say - what are you going to do - what's going to happen?" ' (10 Male)

### Work modifications: assistance from employers/managers

As we have described, Occupational Health had played a limited role in modifying work for the participants in this study. Day to day management of the employee therefore largely rested with the worker themselves, their colleagues and their supervisors or line managers. It was common for the interviewees to talk about support of their colleagues, but perceptions and experiences of managers were mixed. Managers were usually responsible for applying the policies and procedures of absence management, including return-to-work interviews, health and safety management and referral to occupational health.

### Help depends on the individual manager

Some participants had received help from their managers in making minor adjustments which had enabled them to remain at work. One 22 year old described how she had recently started work as a member of a small team in the postal department of a large company before her symptoms became more troublesome. She had changed from her normal occupation (fashion design) due to a combination of stress and back pain, where modifications had been unavailable. Her manager and colleagues in this new job had agreed that when she was in more discomfort they would take a greater share of the heavier manual handling tasks, and were happy for her to take more of their share of computer-based tasks in return. This informal and flexible arrangement had enabled her to feel productive rather than a burden to her colleagues. Similarly, a librarian described how temporary work adjustments agreed with her manager had meant that she was able to reciprocate:

'Well it's a team effort really - I'm doing things that other people aren't. If anybody needs to do my allocation of shelving I'm doing something for them in return.' (21 Female)

However, if duties were reduced indefinitely, with no extra cover, workers might feel that they were burdening their colleagues. There were doubts as to how long their colleagues support might continue. This participant had injured her back at work, and modified duties had been arranged, but she felt that she was not fulfilling her part in the team:

'I feel as though I'm useless. I just poodle about doing what I can, where I can. And the men go "Oh, bloody come out the way" if I try and do something. But they're not going to carry on doing that are they?' (24 Female)

This sense might be heightened by the possibility that their colleagues could question the validity of their pain:

'I know this manager will understand, but because we work as a team its like - do they think I'm swinging the lead? But it's like letting the team down, because you want to be able to do your quota, not put more strain on the other side of the team.' (15 Male)

Inability or unwillingness of employers to address low staffing levels could limit attempts to modify their workload. For this participant, low staffing levels were compounded by a culture that made it difficult to ask for modifications in the form of postural changes. This participant had to maintain an uncomfortable sitting position:

'During the day, can you get up and move if you wanted to?'

'Oh yeh, if I wanted to yeh, but it's the pressure of sort of having to - if I was to do that it'd be "where are you going? You've got work to get done, you ain't got time to go talking' and stuff like that!" "Oh I don't mind you moving around, but you get those ten units done for the post". So you've still got to sit still.'

Although he felt that his employers would be more amenable to modifying his job through the purchase of equipment:

'Oh there'd be no problem in buying a different chair - they're very good like that.' (19 Male)

Whereas other office-based employees described receiving workstation assessments and modifications, this participant had not found her employers (a multinational company) at all helpful in providing her with suitable display screen equipment:

'They don't like to spend money where they think they can get away with it. I mean the chair - I just really, really forced the issue, because I said to the manager - I just can't cope with coming to work, sitting in a chair that's causing me more pain when I get home. And even now, the lap top - this is not the one I originally had - and I did say to them when the other one broke down - maybe now I'll get the monitor and a keyboard separate - "Oh well, we've got a spare one floating round at xxxxx Road, you'll have to have that lap top".'(25 Female)

This office worker again had experienced little support from his manager:

'I did inform the boss about it and - because one time I was lifting from the ground and I felt something jolt in my back and I was in agony and he just said "well why didn't you report straight away?" - because I didn't do that and - he was trying to blame me, that I'd done it elsewhere - "it's not our fault".' (7 Male)

It appeared to be his reduced hours, rather than modified work that had enable him to remain in work:

'I haven't been off sick with my back - because I do part-time anyway so I try and take it easy during the day - and then I can keep at work.' (7 Male)

Whereas this participant's employers had agreed to her taking regular breaks from sitting which had not affected her productivity:

'Generally sitting does cause a problem, although I've got a special chair, and wrist rests and arm rests and foot rests! And I do get up every hour and walk round, and every half hour when it's really painful. Everybody's aware of why I'm doing it. Even though I'm away from my desk for 15-20 minutes an hour, I'm still exceeding my targets. So it's not impacting - I mean people who are there at their desk all the time are not hitting their targets so - I'm constantly exceeding mine.' (4 Female)

### May be over-cautious in their support

Some managers could be overprotective, perhaps due to a sense of responsibility and their own anxieties about back pain, and encourage participants to modify their workload. Participants reacted differently in these situations; some seemed relieved that their problems were being taken account of:

'They've been very good. My immediate manager has been excellent. He's been very good. If I go in and say I can't manage it, it's "well, leave it then".' (24 Female)

While others were less inclined to accept:

'She is very good. She says to me today "what are you carrying that ladder for?" I said - "feel the ladder, it's a lightweight ladder, two step". She says "Oh but you shouldn't have been carrying it". I says "I've got to do my job, you've got to let me do my job. If I can't do my job, there's no point in my being here".' (26 Female)

Lack of adequate help in effective work modifications could lead to further sickness absence, even with the best of intentions. Another participant had been signed off for six weeks following a previous attempt to remain at work on 'light' duties, which failed when he went straight back to his usual duties without a gradual return.

### Managers with experience of back pain perceived to be more sympathetic

As back pain is a common health condition, it is quite likely that managers will also have some experience of back pain. Participants generally felt that their manager was more sympathetic as a result of their own experience of pain:

'I spoke to my boss - he said "yes, take it easy". My boss, he's got long term back pain, and last time he was off with his back he had to wear a support belt and everything, and he understands what it's all about.' (3 Male)

There was a sense that other managers may not be as tolerant of workers with back pain:

'I'm lucky that my line manager he has a back problem as well so he's knows what I go through.' (4 Female)

### Work modifications: patient control

#### Easier to modify workload if in control

In this study some participants were able and/or had chosen to modify their own duties and/or hours on an informal basis, either by themselves, or by involving their colleagues.

This council worker, with a long history of back pain had pursued a combination of self management, taking a few days off work and accessing private manual therapy to remain in work. The nature of his job meant that he was able to adjust his tasks and workload to remain at work most of the time:

'As I say, I have flare-ups, but because I manage my own day, if it ain't the best then I'll stay in the office all day. It just means I'm not climbing in and out of vans all day.' (15 Male)

The ability to modify his workload was also a key factor in work retention for this finance consultant. His flare-ups were now becoming more frequent, but he had been able to manage these by working flexibly:

'No I haven't taken sick leave. I work with it in the sense that say, well, I can't get into the car, go to the office, go up the stairs, I will stay here, do some calculating, phone calls. So that's how I manage it. You could argue that the way I work is self-employed.' (23 Male)

Some participants reported quite minor alterations to their working methods that had helped them to manage the more physically demanding parts of their jobs, as this care assistant describes:

'So I do alter the way I do that a little bit. If I'm moving footplates on wheelchairs, everybody else just bends over - I actually get down on the floor on my knees, and they've provided me with a kneeling pad so I'm not hurting my knees on the floor.' (5 Female)

This building trade worker also described how he had been able to slow down his pace of work:

'I take me time more. I used to go like a bull at a gate, so now I take me time a lot more. It has helped me back. Other than that, nothing else has changed.' (20 Male)

#### The pros and cons of working for oneself

However, working for an unsympathetic boss, and the inability to control his workload had led this participant to start up his own business:

'I'm going to pace meself with what jobs I'm doing. Not take me time as such, cos I'm always used to working at nine hundred mile an hour - but I'll be able to limit meself - do a couple of jobs a day instead of six, seven, eight jobs a day.' (22 Male)

Other participants agreed that there were advantages to being self-employed. One participant with a three year history of back pain had been working part-time as a freelance IT consultant, as well as running his own property development company. Because of difficulty managing his back pain, he had given up the consultancy in order to concentrate on the latter. Although he was worse off financially, it meant that he was able to have more control over his daily routine, and delegate to his employees.

'If I hadn't been self-employed - because of the property business that I've got - but if I was actually working for somebody - I'd probably be unemployed by now.' (2 Male)

#### Fewer options if working alone

Another participant worked in catering both privately, and for an agency. To some extent she could choose how much work to take on, however once she had accepted a booking she was generally working alone without the possibility of adjustments. She considered that asking clients for help would have lessened their confidence in her ability to complete the job.

'When you're actually doing a job and you're doing a good job, if you let them start seeing well I'll have to sit down for five minutes they'll think -" oh she's not very reliable, we won't book her again we'll get somebody else".' (14 Female)

### Colleague support

Others were able to ask colleagues for help on an informal basis when their symptoms were more troublesome. This seemed to work well when the help was available from a team, as this participant describes:

'Oh they're very good. If there's days when I can't bend down - or I sit there in the chair like this - they do things for me.' (11 Female)

However for those who worked with just one other colleague, such informal arrangements appeared to carry greater risks to job retention:

*'I've always gone to work, and who I work beside has been brilliant - you know when my back's been playing up he'll say don't lift those up, I'll do that*. *If he wasn't there I wouldn't be able to do it.' (17 Female)*

## Discussion

Although most people who experience back pain remain at work, or return to work within a few weeks, we do not know if they are successfully managing their duties. Some may work at reduced capacity, rely on the help of colleagues, remain on adjusted duties or hours, have periods of absence for a secondary illness such as depression, take early retirement, or change occupation. If modifications are unavailable or ineffective, healthy and productive work may prove unsustainable.

In this study, we found that most participants were making informal modifications to help them remain at work, either independently or jointly with their colleagues and line manager. Those who could manage their own workload or a choice of tasks had an obvious advantage. Some of these modifications were simple, and used flexibly when the need arose.

Only a minority of the participants in this study had received support through occupational health services, often following a period of sickness absence. Self-referral was unusual. Experiences of occupational health varied; modifications may not have been considered or have been inappropriate or ineffective. Implementation largely rested with the line manager; there were few examples of face-to-face communication between all the parties concerned, leaving the details to the interpretation of the manager and the employee. Those whose symptoms had followed a workplace accident seemed to have received more attention, perhaps due to employers' fears of compensation claims. Such concerns may lead to an over-cautious approach.

In the UK only a minority of the workforce can access occupational healthcare compared with countries within the European Union. In 2006 the Faculty of Occupational Medicine of the Royal College of Physicians reported that some member states (e.g. the Netherlands, France, Belgium, and Finland) achieve 90% coverage [19]; UK coverage was estimated at 34%. Only Greece at 28% had lower coverage, although the nature of services differs between the states. In the UK the extent of the service is determined by the costs that employers are willing/able to bear. Employees' perceptions of the confidentiality and affiliation of occupational health are also important. Reducing the number of sickness absence days that 'trigger' a referral to occupational health may lead to more effective management of musculoskeletal disorders [20], however if the service is viewed solely in connection with disciplinary procedures, employees may be reluctant to access it. Previous UK research suggests that the implementation of occupational health guidelines, particularly prompt intervention, may be hindered by organisational obstacles such as strict referral criteria [21].

Line managers have a vital role in supporting employees with health conditions such as low back pain. Their beliefs and attitudes, and the support and guidance available to them, can either facilitate or impede the employee. A recent study of line manager competencies [22] recognises that line managers are 'the key to work adjustments and implementation of work redesign initiatives' and that they require support in this role. The report concludes that managers do not need to be knowledgeable about health conditions to be effective, however, it would seem from our findings that some basic understanding of pain mechanisms may be helpful in clarifying whether tasks are 'harmful' to the back.

In this study, participants considered that managers were more sympathetic if they had also experienced back pain. However, sympathy in itself did not necessarily lead to appropriate management. If a manager believes that pain should be avoided, and that heavy work is inherently dangerous, their approach may be overcautious and result in permanent restrictions. The ease with which work modifications can be made has been described as 'adjustment latitude' [23]. There is a risk however, that if workers are able to, and choose to avoid certain tasks because they think they are unsafe or will make their condition worse, then it may become a permanent arrangement and lead to reduced capacity. These 'representations' (thoughts, attitudes and beliefs) that an individual has of their condition are one of the key factors in the 'margin of manoeuvre' model described by Durand et al [24]. The findings of our study suggest that the representations held by managers and other stakeholders are also important. Much research has studied the effect of fear-avoidance beliefs of patients, GPs and other clinicians [25,26]. Those of employers, line managers and work colleagues are of equal importance, but feature less in the literature.

In our study, participants' experiences of line managers were mixed. In her study, Foster concluded that 'employees are reliant upon the goodwill of individual line managers for successful adjustments, turning what should be a legal obligation into a personal lottery' [27]. Research conducted for the British Occupational Health Foundation [22] found that the relationship with the manager prior to sickness absence had a bearing on return to work, and suggested that the attitudes of managers were perceived by employees as varying according to the health condition. In a study of university employees, Munir et al [28] found that only 50% of those with chronic health conditions had disclosed their condition to their boss. Employees with back pain who are concerned about being seen as fraudulent or unreliable may be unwilling to disclose their condition [16]. Their need to maintain an identity of independence and ability, and/or not wanting to appear pre-occupied with their pain may be a barrier to seeking support [29] and they may perceive themselves as primarily responsible for managing their condition at work [22,30]. However, interventions designed to empower employees with chronic diseases suggest that it is possible to train employees to negotiate work accommodations [31].

Modifications should be temporary and involve a gradual return to full hours and duties. However the line manager may then be faced with conflicting demands if productivity levels are subsequently reduced. The effect on other workers also has to be considered. The participants in our study did not want to be a 'burden' to their colleagues, and felt more comfortable about receiving help if they were able to reciprocate in some way. Some workplaces are better able to offer modifications than others due to staffing levels and the variety of work tasks. Other research has shown that fewer options are available when the work is highly specialised, or physically demanding [32]. It may be difficult for employers to see the long term 'business case' for offering modifications and as organisations become 'leaner' there is a risk that lower staffing levels increase individual workloads with fewer options for adjustment.

In our study, where modifications had been made by employers, more attention seemed to be paid to adjusting equipment, such as seating, rather than grading tasks, such as lifting, which were more likely to be avoided. Employees need to be able to consider a wide range of different types of modification, but the study by Foster [27] concluded that managers were more likely to favour the provision of equipment, rather than adjustments to work itself that might involve changes to employment conditions.

### Limitations and strengths

This was a convenience sample of employed patients who had been referred for back pain rehabilitation. The majority had taken sick leave, some for several weeks. The participants may therefore not be representative of those who are managing their back pain more successfully at work and so limit the extent to which the findings can be generalised to a wider population. However most participants did work for large employers and might have been expected to have received greater involvement from Occupational Health in work modification. There were comparatively fewer participants recruited who were self-employed or working in small or medium sized enterprises (<250 employees). This may reflect the UK economy where although small enterprises (<50 employees) account for the majority of businesses, large employers (public and private) account for the majority of the workforce. Alternatively it may be that the pressures of working for a small employer impose actual or perceived obstacles to accessing healthcare and taking part in a research study.

## Conclusions

In this study, work modifications seemed to be mainly casual arrangements made between the employee, line manager and colleagues. Occupational health was usually involved only after substantial work absence. There appeared to be insufficient expertise among managers and occupational health in adjusting work, little indication of joint planning, and overcautious approaches were common.

## Competing interests

The authors declare that they have no competing interests.

## Authors' contributions

CC, PJW and AD designed the study. CC conducted and transcribed the interviews and drafted the manuscript. CC conducted the initial data coding and analysis. PJW checked and reviewed the coding and analysis. PJW and AD revised the manuscript. All authors read and approved the final manuscript.

## Pre-publication history

The pre-publication history for this paper can be accessed here:

http://www.biomedcentral.com/1471-2474/11/277/prepub
